# Fruit-Fly-Optimized Weighted Averaging Algorithm for Data Fusion in MEMS IMU Array

**DOI:** 10.3390/mi16070739

**Published:** 2025-06-24

**Authors:** Ting Zhu, Gao Peng, Jianping Li, Jiawei Xuan, Jingbei Tian

**Affiliations:** 1 School of Automation, Guangxi University of Science and Technology, Liuzhou 545006, China; t@imu.wiki (T.Z.); 221077077@stdmail.gxust.edu.cn (G.P.); 20230201015@stdmail.gxust.edu.cn (J.X.); 2005090@gxust.edu.cn (J.T.); 2 Beijing Institute of Control Engineering, Beijing 100094, China

**Keywords:** inertial measurement, MEMS IMU array, data fusion, weighted averaging algorithm, fruit fly optimization algorithm

## Abstract

The weighted averaging algorithm is a widely adopted high-efficiency data fusion approach for micro-electro-mechanical system (MEMS) inertial measurement unit (IMU) array, where the configuration of weighting coefficients plays a critical role in improving measurement accuracy. In this study, an optimal weighted averaging algorithm based on the fruit fly optimization algorithm (FOA) is proposed by analyzing the data fusion mechanism of the MEMS IMU array. Firstly, a measurement model for the MEMS IMU array is constructed, and the principles of data fusion are systematically investigated. Secondly, the optimal weighting coefficients under ideal conditions are derived, and their limitations in practical applications are discussed. Building on this framework, the FOA is employed to search for optimal weights, enabling the realization of high-precision weighted averaging fusion. Simulation and experimental results demonstrate that the proposed method outperforms conventional approaches in terms of accuracy and robustness.

## 1. Introduction

Micro-electro-mechanical system (MEMS) inertial measurement units (IMUs) have been widely adopted in unmanned aerial vehicle navigation, smart wearable devices, and robotic positioning due to their compact size, low cost, and low power consumption [1,2]. However, constrained by current technological limitations in MEMS manufacturing processes, MEMS IMUs demonstrate significantly higher errors compared to conventional optical or mechanical IMUs, particularly manifesting in parameters such as bias instability and scale factor errors. The presence of these errors will make the IMU unable to meet high-precision navigation requirements when a single IMU operates independently. Recent studies have demonstrated that multi-IMU array data fusion techniques serve as an effective approach to enhance overall system performance [3].

The IMU array technology was first proposed by Bayard et al., JPL Laboratory in 2003 [4]. Bayard uses MEMS gyro as the research object to compose a gyro array, and the performance of the array is improved by 173 times after data fusion. Since 2008, Tanenhaus and Aaaociates have released a number of low-cost and high-precision inertial navigation systems based on multi-MEMS gyros and accelerometers. One of the more representative is the three-axis IMU composed of 24 MEMS gyros and 6 accelerometers, and the 5 h angular drift of this product can be better than 0.03° [5,6,7]. In 2015, Wang et al. at the University of Michigan assembled 72 MEMS gyroscopes on a three-layer development board and proposed a principled algorithm that exploits non-linear dynamics which reduces the angle error by about 50% compared to the Mean and Kalman algorithms [8]. In 2019, Owais et al. proposed an IMU array data acquisition and data fusion architecture based on Artix-7 series low-cost FPGA, marking that IMU array research has entered the application stage [9,10]. In 2024, the MEMS IMU array was widely adopted across diverse applications. Zhang proposed a robust and efficient INS-level fusion algorithm for IMU arrays/GNSS that maintains computational efficiency comparable to IMU-level fusion while achieving the robustness of INS-level fusion [11]. Lin proposed a pedestrian dead reckoning (PDR) method using an array of four consumer-grade MEMS IMUs with a weighted data fusion algorithm, achieving a 0.5% horizontal positioning error and reducing errors and noise by 0.9 times compared to a single IMU [12].

The data fusion algorithm is the key to suppressing random noise and improving the measurement accuracy of the MEMS IMU array. In the study of the gyro array, Bayard uses a classical Kalman filter to fuse the output signal [4]. With the increasing array size, many new fusion algorithms have been applied to MEMS IMU arrays, such as the combined filtering scheme [13], hidden Markov model [8], maximum likelihood estimation algorithm [14], optimal linear combination method [15] and LSTM-Kalman [16]. However, with the increase in array scale, the filtering-based data fusion method consumes more and more computing resources, which greatly affects real-time data processing. In practical engineering applications, the weighted averaging algorithm has become one of the mainstream methods because of its simple implementation and high computational efficiency [17,18,19,20]. Current research indicates that the optimal allocation of weighting coefficients constitutes a critical factor influencing array fusion effectiveness. Conventional methods predominantly employ static weight allocation strategies based on variance reciprocals or empirical formulas, which have certain limitations. With the rapid development of intelligent optimization algorithms, it has become possible to further optimize weight allocation. Among them, the fruit fly optimization algorithm (FOA), as an easily implementable swarm intelligence algorithm, has shown unique advantages in the field of parameter optimization [21,22].

In this study, a weighted averaging fusion method based on improved FOA is proposed to construct a weight optimization model for the MEMS IMU array. The remainder of this paper is structured as follows. First, in Section 2, the errors of MEMS IMU are modeled and analyzed, and the data fusion mechanism of the MEMS IMU array is studied. Second, the weighted averaging algorithm of data fusion in MEMS IMU array is studied, and the weighted averaging algorithm based on improved FOA is proposed in Section 3. The proposed method has been applied to a MEMS IMU array prototype, and then simulation and experiment are discussed in Section 4. Finally, Section 5 presents the conclusions of this study.

## 2. Error Analysis and Data Fusion Mechanism of MEMS IMU Array

### 2.1. Error Modeling and Analysis of MEMS IMU

The errors of MEMS IMU are usually modeled by bias, noise, scale factor errors, installation errors, etc. The inertial sensor of MEMS IMU mainly includes the gyroscope and accelerometer, and the sensor errors are the difference between the measured value of the gyroscope or accelerometer and the real value.

Taking the gyroscope as an example, the relationship between the true value and the measured value can be expressed as follows:

(1) $$N^{g}=K^{g}\omega^{g}+b^{g}+\eta^{g}$$

where $\omega^{g}$ represents the true angular velocity acting on the IMU, $N^{g}$ represents the measured value of the gyroscopes, $b^{g}$ represents the bias of the gyroscopes, $\eta^{g}$ represents the noise of the gyroscopes, $K^{g}$ includes the scale factor errors and installation errors of the gyroscopes.

Similarly, the relationship between the true value and the measured value can be expressed as follows:

(2) $$N^{a}=K^{a}f^{a}+b^{a}+\eta^{a}$$

where $f^{a}$ represents the true specific force acting on the IMU, $N^{a}$ represents the measured value of the accelerometers, $b^{a}$ represents the bias of the accelerometers, $\eta^{a}$ represents the noise of the accelerometers, $K^{a}$ includes the scale factor errors and installation errors of the accelerometers.

The constant errors of MEMS IMU can be compensated by calibration, such as bias, scale factor errors, and installation errors. However, random errors cannot be compensated, such as noise, which will have a great impact on the navigation accuracy of the system.

**Figure 1:**
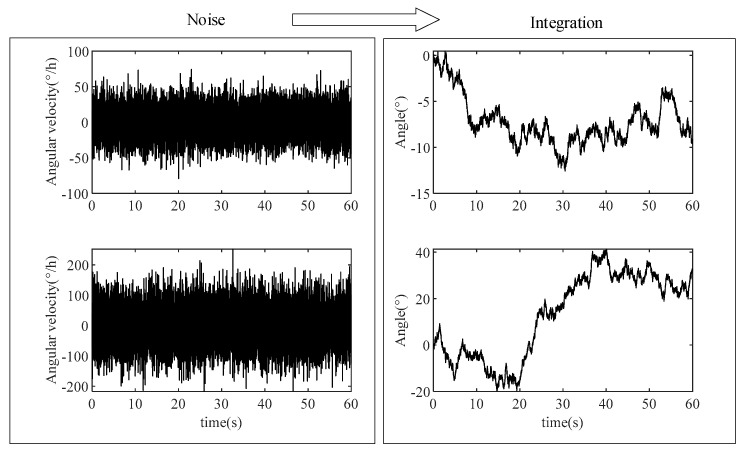
The influence of gyroscopic noise on navigation calculation. Here, angular velocity noise is simulated by Gaussian white noise, and its influence on navigation calculation is simulated by angular velocity noise integral.

Taking the gyroscope as an example, we use Gaussian white noise to simulate the noise of two gyroscopes with different amplitudes. The random walk of angles caused by angular velocity noise with different amplitudes after navigation calculation is shown in Figure 1.

From the pure angular velocity noise integration, it can be seen that this integration will cause a random walk of angle. The larger the noise amplitude, the larger the angular random walk amplitude. Angular velocity integration is one of the main steps in navigation calculation, which will have a great impact on navigation accuracy. The random errors of gyroscopes are far greater than the pure noise, so the navigation error caused by them will be greater.

**Figure 2:**
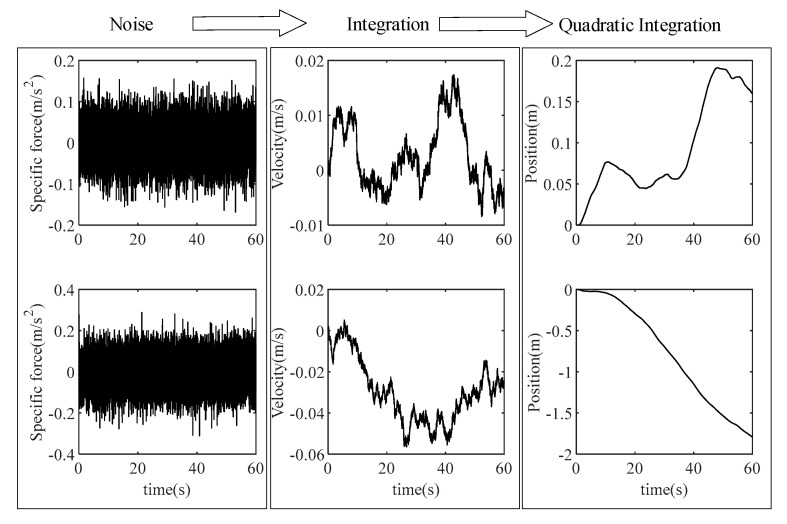
The influence of specific force noise on navigation calculation. Here, specific force noise is simulated by Gaussian white noise, its influence on velocity is simulated by the first integration of the specific force noise, and the influence of the position is simulated by the quadratic integration of the specific force noise.

Similarly, a random walk of velocity is caused by specific force noise after navigation calculations, and then a fast divergence of position is caused, as shown in Figure 2.

From the pure specific force velocity noise integration, it can be seen that this integration will cause the random walk of velocity and the divergence of position. The larger the noise amplitude, the larger the amplitude of the velocity random walk, and the larger the divergence of the position. The first and quadratic integrations of the specific force are the main steps in navigation calculation, which will have a great impact on navigation accuracy. The random errors of the accelerometer are far greater than the pure noise, so the navigation error caused by them will be greater.

From the above analysis of the influence of gyroscope and accelerometer noise on navigation accuracy, it can be seen that the suppression of random error plays an important role in improving navigation accuracy. Therefore, the suppression of MEMS IMU random error is very necessary.

### 2.2. Data Fusion Mechanism of MEMS IMU Array

To improve measurement accuracy, MEMS IMUs are combined in the form of horizontal and vertical arrays to construct IMU arrays, and random errors are suppressed by data fusion. A triaxial orthogonal coordinate frame is established for the IMU array, named the s-frame, and the coordinate frame of each IMU overlaps with the s-frame.

Considering angular velocity as an example, the conversion equation from the s-frame to gyroscopes without errors is

(3) $$\omega^{g}=H^{g}\omega^{s}$$

where $\omega^{g}$ is the angular velocity vector of gyroscopes, which may be expressed as follows:

(4) $$\omega^{g}={\left[\begin{array}{c} \omega_{1x}^{g}\\ \omega_{1y}^{g}\\ \omega_{1z}^{g}\\ \vdots \\ \omega_{Nx}^{g}\\ \omega_{Ny}^{g}\\ \omega_{Nz}^{g} \end{array}\right]}_{3N\times 1}$$

where *N* represents the number of IMUs in the array, and *x*, *y*, *z* represent the three orthogonal axes in the IMU.

Additionally, $\omega^{s}$ is the angular velocity vector in the s-frame, and it may be expressed as follows:

(5) $$\omega^{s}=\left[\begin{array}{c} \omega_{x}^{s}\\ \omega_{y}^{s}\\ \omega_{z}^{s} \end{array}\right]$$

**Figure 3:**
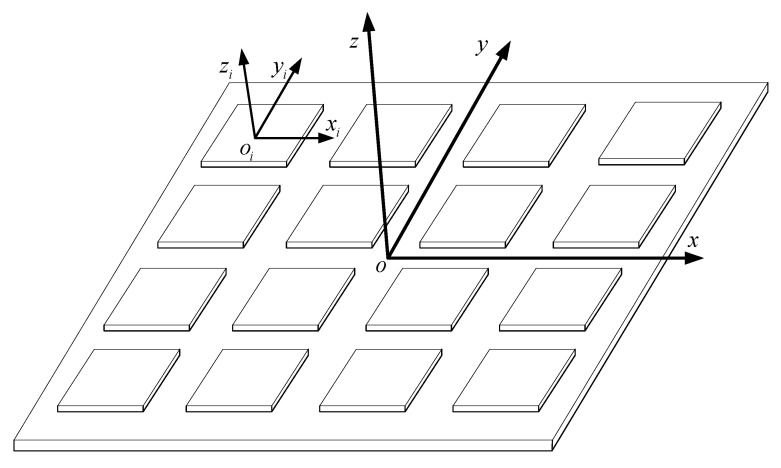
The structure of the MEMS IMU array.

Finally, $H^{g}$ is the IMU array configuration matrix. The structure of the IMU array is shown in Figure 3, and the configuration matrix is

(6) $$H^{g}={\left[\begin{array}{ccc} 1 & 0 & 0\\ 0 & 1 & 0\\ 0 & 0 & 1\\ \vdots & \vdots & \vdots \\ 1 & 0 & 0\\ 0 & 1 & 0\\ 0 & 0 & 1 \end{array}\right]}_{3N\times 3}$$

We can then obtain $\omega^{s}$ via the least squares (LS) method as follows:

(7) $$\omega^{s}={\left(H^{gT}H^{g}\right)}^{-1}H^{gT}\omega^{g}$$

Combining Equations (6) and (7), $\omega^{s}$ can also be expressed as follows:

(8) $$\omega^{s}=\frac{1}{N}\left[\begin{array}{c} \sum_{i=1}^{N}\omega_{ix}^{g}\\ \sum_{i=1}^{N}\omega_{iy}^{g}\\ \sum_{i=1}^{N}\omega_{iz}^{g} \end{array}\right]$$

It can be seen that by averaging the output data of each gyroscope in the array, the angular velocity data fusion in the MEMS IMU array can be realized.

Similarly, the specific force in the s-frame can also be obtained by fusion of the specific force measurements of each accelerometer:

(9) $$f^{s}=\frac{1}{N}\left[\begin{array}{c} \sum_{i=1}^{N}f_{ix}\\ \sum_{i=1}^{N}f_{iy}\\ \sum_{i=1}^{N}f_{iz} \end{array}\right]$$

Bias stability is an important parameter to characterize the measurement accuracy of IMU, which is obtained by collecting the output value of IMU at a static state and calculating its standard deviation. Taking the *x*-axis angular velocity of the IMU array as an example, if the bias stability of all gyros along this axis is consistent, the bias stability of the *x*-axis angular velocity of the array can be expressed as follows:

(10) $$\sigma_{x}^{s}=\sqrt{\mathrm{var}(\frac{1}{N}\sum_{i=1}^{N}\omega_{ix}^{g})}=\sqrt{\frac{1}{N^{2}}\sum_{i=1}^{N}\mathrm{var}(\omega_{ix}^{g})}=\sqrt{\frac{1}{N^{2}}\sum_{i=1}^{N}\sigma_{x}^{g}}=\frac{\sigma_{x}^{g}}{\sqrt{N}}$$

where var represents the variance of the calculation, $\sigma_{x}^{g}$ represents the bias stability of each gyroscope in the *x*-axis.

**Figure 4:**
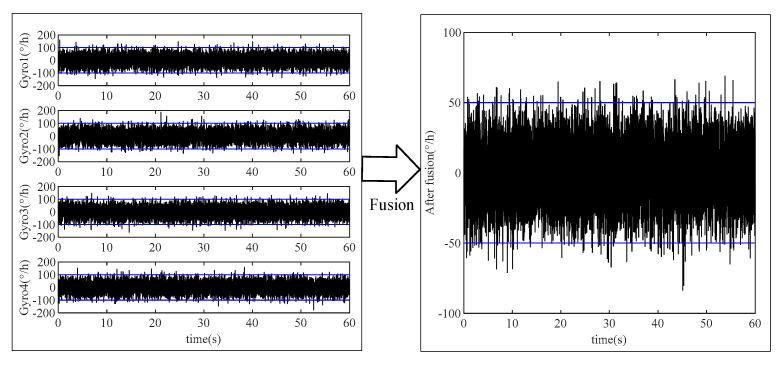
The comparison before and after data fusion.

Similarly, it can be seen that if the accuracy of gyroscopes and accelerometers in each axis of the IMU array is consistent, the measurement accuracy of the array will be improved by $\sqrt{N}$ times when the average method is used for data fusion. The comparison of before and after data fusion is shown in Figure 4.

The above simulation takes the gyroscope noise as an example and fuses the data of four gyros. It can be seen that the gyro noise amplitude after data fusion is about half of the original, which confirms the effectiveness of data fusion.

## 3. Fruit-Fly-Optimized Weighted Averaging Algorithm

### 3.1. Weighted Averaging Algorithm for Data Fusion

It can be seen from Section 2 that the averaging method has a good data fusion effect when the sensor precision is the same. However, in practical applications, the accuracy of each sensor is often inconsistent. This case will reduce the average method of data fusion effect. A good way is to configure different sensors by weights.

According to Equation (7), we can then obtain $\omega^{s}$ via the weighted least squares (WLS) method as follows:

(11) $$\omega^{s}={\left(H^{gT}W^{g}H^{g}\right)}^{-1}H^{gT}W^{g}\omega^{g}=\left[\begin{array}{c} \frac{\sum_{i=1}^{N}w_{ix}^{g}\omega_{ix}^{g}}{\sum_{i=1}^{N}w_{ix}^{g}}\\ \frac{\sum_{i=1}^{N}w_{iy}^{g}\omega_{iy}^{g}}{\sum_{i=1}^{N}w_{iy}^{g}}\\ \frac{\sum_{i=1}^{N}w_{iz}^{g}\omega_{iz}^{g}}{\sum_{i=1}^{N}w_{iz}^{g}} \end{array}\right]$$

where $W^{g}$ represents the weight of each gyroscope in the data fusion, as follows:

(12) $$W^{g}=\mathrm{diag}{\left[\begin{array}{ccccccc} w_{1x}^{g} & w_{1y}^{g} & w_{1z}^{g} & \ldots & w_{Nx}^{g} & w_{Ny}^{g} & w_{Nz}^{g} \end{array}\right]}_{3N}$$

Similarly, the specific force in the s-frame can also be obtained as follows:

(13) $$f^{s}={\left(H^{aT}W^{a}H^{a}\right)}^{-1}H^{aT}W^{a}f^{a}=\left[\begin{array}{c} \frac{\sum_{i=1}^{N}w_{ix}^{a}f_{ix}^{a}}{\sum_{i=1}^{N}w_{ix}^{a}}\\ \frac{\sum_{i=1}^{N}w_{iy}^{a}f_{iy}^{a}}{\sum_{i=1}^{N}w_{iy}^{a}}\\ \frac{\sum_{i=1}^{N}w_{iy}^{a}f_{iy}^{a}}{\sum_{i=1}^{N}w_{iy}^{a}} \end{array}\right]$$

where $W^{a}$ represents the weight of each accelerometer in the data fusion and $H^{a}$ is the IMU array configuration matrix, as follows:

(14) $$\left\{\begin{array}{l} W^{a}=\mathrm{diag}{\left[\begin{array}{ccccccc} w_{1x}^{a} & w_{1y}^{a} & w_{1z}^{a} & \ldots & w_{Nx}^{a} & w_{Ny}^{a} & w_{Nz}^{a} \end{array}\right]}_{3N}\\ H^{a}=H^{g} \end{array}\right.$$

It can be seen that the WLS is equivalent to the weighted averaging algorithm for the data fusion of the MEMS IMU array. If only the measurement noise is considered as the error source, it can be proven that the optimal weight is

(15) $$W=R^{-1}$$

where ***R*** is the noise variance of Gaussian white noise.

When $R^{-1}$ is used as the weight for data fusion, Equations (11) and (13) are also called Markov estimation. It can be seen that Markov estimation is the optimal weighted averaging algorithm when only Gaussian white noise is considered. However, the actual MEMS IMU noise is not Gaussian white noise. In addition to noise, MEMS IMU also has other random errors, such as random walks of angular velocity. Therefore, Markov estimation is not the optimal weighted averaging algorithm in practical applications, and the corresponding weights need to be configured according to the actual data.

### 3.2. Design of Optimal Weighted Averaging Algorithm Based on FOA

It is difficult to estimate the weight of the weighted averaging algorithm in MEMS IMU array data fusion using error analysis or mathematical derivation. The development of swarm intelligence algorithms provides a new method for weight optimization. As a new swarm intelligence algorithm, the fruit fly optimization algorithm (FOA) has the characteristics of fast convergence and low computing power. In this study, the optimal weight of weighted averaging in MEMS IMU array data fusion is estimated based on FOA.

First, the output data of the MEMS IMU array is collected in a static state. The random error is retained by subtracting the mean value, as follows:

(16) $$\left\{\begin{array}{l} {\tilde{\omega }}^{g}=\omega^{g}-{\bar{\omega }}^{g}\\ {\tilde{f}}^{a}=f^{a}-{\bar{f}}^{a} \end{array}\right.$$

where ${\bar{\omega }}^{g}$ represents the average of all data collected by each gyroscope, ${\tilde{\omega }}^{g}$ represents the random error of each gyroscope, ${\bar{f}}^{a}$ represents the average of all data collected by each accelerometer, and ${\tilde{f}}^{a}$ represents the random error of each accelerometer.

Then, the weighted averaging algorithm is used to fuse the gyroscope and accelerometer data of the corresponding axis, respectively. After fusion, the random errors of the angular velocity and specific force measurements of the MEMS IMU array are shown as follows:

(17) $${\tilde{\omega }}^{s}=\left[\begin{array}{c} {\tilde{\omega }}_{x}^{s}\\ {\tilde{\omega }}_{y}^{s}\\ {\tilde{\omega }}_{z}^{s} \end{array}\right]=\left[\begin{array}{c} \frac{\sum_{i=1}^{N}w_{ix}^{g}{\tilde{\omega }}_{ix}^{g}}{\sum_{i=1}^{N}w_{ix}^{g}}\\ \frac{\sum_{i=1}^{N}w_{iy}^{g}{\tilde{\omega }}_{iy}^{g}}{\sum_{i=1}^{N}w_{iy}^{g}}\\ \frac{\sum_{i=1}^{N}w_{iz}^{g}{\tilde{\omega }}_{iz}^{g}}{\sum_{i=1}^{N}w_{iz}^{g}} \end{array}\right]$$

(18) $${\tilde{f}}^{s}=\left[\begin{array}{c} {\tilde{f}}_{x}^{s}\\ {\tilde{f}}_{y}^{s}\\ {\tilde{f}}_{z}^{s} \end{array}\right]=\left[\begin{array}{c} \frac{\sum_{i=1}^{N}w_{ix}^{a}{\tilde{f}}_{ix}^{a}}{\sum_{i=1}^{N}w_{ix}^{a}}\\ \frac{\sum_{i=1}^{N}w_{iy}^{a}{\tilde{f}}_{iy}^{a}}{\sum_{i=1}^{N}w_{iy}^{a}}\\ \frac{\sum_{i=1}^{N}w_{iy}^{a}{\tilde{f}}_{iy}^{a}}{\sum_{i=1}^{N}w_{iy}^{a}} \end{array}\right]$$

The purpose of MEMS IMU data fusion is to suppress random errors. For actual random errors, the improved FOA is used to estimate the optimal weight of the weighted averaging algorithm based on historical data. The operation steps are as follows:M

(1) Firstly, the weight of the *x*-axis gyroscopes weighted averaging data fusion is estimated.
(2) Construct fruit flies in N-dimensional space and initialize fruit-fly group position:
  (19) $$\left[\begin{array}{c} D_{1}\\ D_{2}\\ \vdots \\ D_{N} \end{array}\right]=\left[\begin{array}{c} Rand(0,1)\\ Rand(0,1)\\ \vdots \\ Rand(0,1) \end{array}\right]$$
  where $\left(D_{1},D_{2},\ldots ,D_{N}\right)$ represents the fruit-fly population position, Rand(0,1) represents random values in the interval (0, 1).
(3) With the group position as the center, individual fruit flies randomly fly out to forage. The individual location of fruit flies is shown as follows:
  (20) $$\left[\begin{array}{c} d_{k1}\\ d_{k2}\\ \vdots \\ d_{kN} \end{array}\right]=\left[\begin{array}{c} D_{1}+Rand(0,1)\\ D_{2}+Rand(0,1)\\ \vdots \\ D_{N}+Rand(0,1) \end{array}\right]$$
  where $\left(d_{k1},d_{k2},\ldots ,d_{kN}\right)$ represents the position of the *k*th individual fruit fly, *k* = 1, 2, …, *K*, *K* is the total number of fruit flies.
(4) Take $\left(d_{k1},d_{k2},\ldots ,d_{kN}\right)$ as the *N* weights of the *x*-axis gyroscopes in Equation (17), calculate ${\tilde{\omega }}_{x}^{s}$ at the corresponding time after fusion according to all-time data collected, and calculate the bias stability of ${\tilde{\omega }}_{x}^{s}$, that is, standard deviation, to build the smell concentration as follows:
  (21) $$Smell_{k}=1/\mathrm{std}\left({\tilde{\omega }}_{x}^{s}\right)$$
  where std means to calculate the standard deviation for ${\tilde{\omega }}_{x}^{s}$ at all times.
(5) Find the individual with the maximum smell concentration, and all fruit flies flock to that location:
  (22) $$\left\{\begin{array}{c} Smell_{best}=\max\left(Smell_{i}\right)\\ \left[D_{1},D_{2},\ldots ,D_{N}\right]=\left[d_{best1},d_{best2},\ldots ,d_{bestN}\right] \end{array}\right.$$
  where $Smell_{best}$ represents the maximum smell concentration, $\left[d_{best1},d_{best2},\ldots ,d_{bestN}\right]$ represents the coordinate of the individual with the maximum smell concentration, and max represents the maximum value.
(6) The total number of fruit flies and the number of iterations are set, and the specific total number of fruit flies and the number of iterations are determined according to the actual case. The total number of iterations can be set to 1000, and generally it takes about 300 iterations to reach the optimal level. To be safe, we have set a total of 1000 iterations. Steps (3) to (5) are repeated for iterative calculation, and the last updated group position is the final optimal position.
(7) Repeat steps (1) to (6) to estimate the optimal weight of ${\tilde{\omega }}_{y}^{s}$, where ${\tilde{\omega }}_{x}^{s}$ is replaced by ${\tilde{\omega }}_{y}^{s}$;
(8) Repeat steps (1) to (6) to estimate the optimal weight of ${\tilde{\omega }}_{z}^{s}$, where ${\tilde{\omega }}_{x}^{s}$ is replaced by ${\tilde{\omega }}_{z}^{s}$;
(9) Repeat steps (1) to (6) to estimate the optimal weight of ${\tilde{f}}_{x}^{s}$, where ${\tilde{\omega }}_{x}^{s}$ is replaced by ${\tilde{f}}_{x}^{s}$;
(10) Repeat steps (1) to (6) to estimate the optimal weight of ${\tilde{f}}_{y}^{s}$, where ${\tilde{\omega }}_{x}^{s}$ is replaced by ${\tilde{f}}_{y}^{s}$;
(11) Repeat steps (1) to (6) to estimate the optimal weight of ${\tilde{f}}_{z}^{s}$, where ${\tilde{\omega }}_{x}^{s}$ is replaced by ${\tilde{f}}_{z}^{s}$.

## 4. Simulation and Experiment

### 4.1. Simulation

To demonstrate the correctness of the proposed weighted averaging algorithm for MEMS IMU array data fusion, a simulation is conducted based on an IMU array with 5 IMUs. In the simulation, the suppression of random errors of MEMS IMU arrays by the proposed method, averaging method, and Markov method is compared. Considering the similarity of the data fusion methods of the gyroscope and accelerometer, only the gyroscope in the IMU is simulated. Due to the limitations of the simulations, it is impossible to simulate all possible errors of the gyroscope. Therefore, only stability is considered in the simulation.

First, the bias stabilities of the simulated gyroscopes are set. With reference to the bias stability of the actual MEMS gyroscope, the bias stabilities of the simulated gyroscopes are set as shown in Table 1.

Second, bias stability is simulated by Gaussian white noise, and the random errors of 5 gyroscopes are generated, respectively. Based on the gyroscope random errors generated by the simulation, the weights of the weighted averaging algorithm are estimated using the proposed method, and the weights obtained are as follows:

(23) $$W_{proposed}^{g}=\mathrm{diag}[\begin{array}{ccccc} 0.50 & 0.23 & 0.13 & 0.08 & 0.06 \end{array}]$$

The Markov method can be expressed by the weighted averaging algorithm, and its weights can be obtained as follows according to Table 1 and Equation (15):

(24) $$W_{Markov}^{g}=\mathrm{diag}[\begin{array}{ccccc} 0.50 & 0.23 & 0.13 & 0.08 & 0.06 \end{array}]$$

The averaging method can be expressed by the weighted averaging algorithm, and its weights can be as follows:

(25) $$W_{ave}^{g}=\mathrm{diag}[\begin{array}{ccccc} 1 & 1 & 1 & 1 & 1 \end{array}]$$

Finally, the weights of these three methods are used to fuse MEMS IMU array data. The bias stability comparison after fusion is shown in Table 2.

The simulation is performed under ideal conditions and the random error is Gaussian white noise. Therefore, the Markov method is the best data fusion method. The proposed method uses swarm intelligence to search for optimization, and obtains the effect equivalent to the optimal method. The simulation results fully demonstrate the correctness of the proposed method.

### 4.2. Experiment

**Figure 5:**
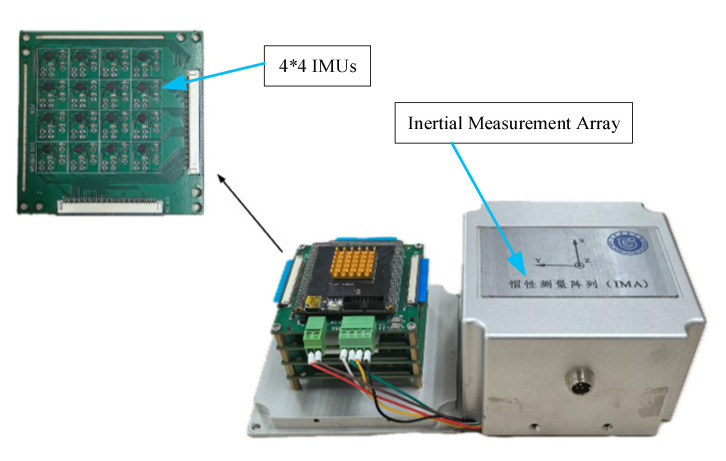
MEMS IMU array prototype.

To demonstrate the effectiveness of the proposed weighted averaging algorithm for MEMS IMU array data fusion, a MEMS IMU array prototype with 16 IMUs is developed and the data fusion experiment is performed. The MEMS IMU array developed is shown in Figure 5, and the MEMS IMU used is MPU-6500 (Manufacturer: InvenSense Inc., San Jose, CA, USA).

**Figure 6:**
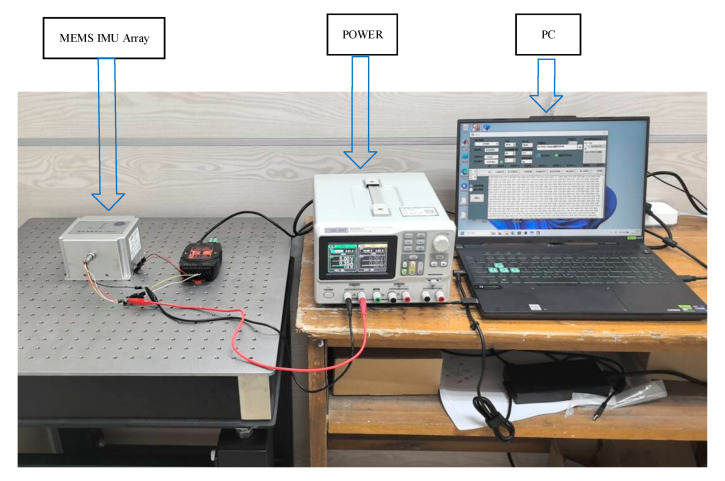
MEMS IMU data fusion experimental setup.

In the experiment, the MEMS IMU array is placed on the static platform, and the output data of the array is collected by a computer for about 1 h. The MEMS IMU data fusion experimental setup is shown in Figure 6.

**Figure 7:**
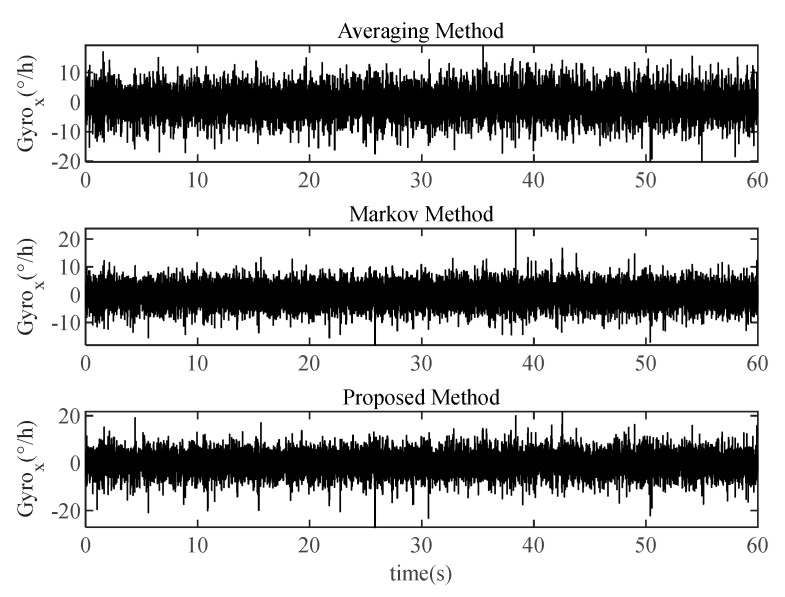
The fused data in MEMS IMU array prototype by three methods, Take the *x*-axis gyroscope for an example.

The averaging method, Markov method, and proposed method are used for data fusion of the output data, and the 60 s *x*-axis gyroscope fused data is shown in Figure 7. Their bias stabilities based on 1 h data are calculated which is shown in Table 3.

It can be seen from the comparison of bias stability after data fusion that the Markov method is better than the averaging method, and the proposed method achieves the best effect among the three methods in the *x*, *y*, and *z* axes. This is because the Markov method is based on Gaussian white noise for weight estimation, while actual sensor noise is often not strictly Gaussian white noise. Therefore, using FOA-based weight optimization can obtain better weights.

To further compare the influence of different data fusion methods on navigation, random errors are extracted from the fused data according to Equation (16), and the navigation errors are calculated as follows:

(26) $$\left\{\begin{array}{l} \dot{\phi }=\phi \times \omega_{in}^{n}+\delta \omega_{in}^{n}-\left(I-\phi \times \right){\tilde{\omega }}^{g}\\ \delta \dot{v}=-\phi \times f^{n}+\delta v\times \left(2\omega_{ie}^{n}+\omega_{en}^{n}\right)+v\times \left(2\delta \omega_{ie}^{n}+\delta \omega_{en}^{n}\right)+\left(I-\phi \times \right){\tilde{f}}^{a}\\ \delta \dot{p}=\delta v \end{array}\right.$$

(27) $$\phi =\left[\begin{array}{c} \phi_{e}\\ \phi_{n}\\ \phi_{u} \end{array}\right],\delta v=\left[\begin{array}{c} \delta v_{e}\\ \delta v_{n}\\ \delta v_{u} \end{array}\right],\delta p=\left[\begin{array}{c} \delta p_{e}\\ \delta p_{n}\\ \delta p_{u} \end{array}\right]$$

where $\phi_{e}$, $\phi_{n}$ and $\phi_{u}$ represent the attitude errors in the east, north, and upward directions, respectively, $\delta v_{e}$, $\delta v_{n}$ and $\delta v_{u}$ represent the velocity errors in the east, north, and upward directions, respectively, $\delta p_{e}$, $\delta p_{n}$ and $\delta p_{u}$ represent the position errors in the east, north, and upward directions, respectively.

**Figure 8:**
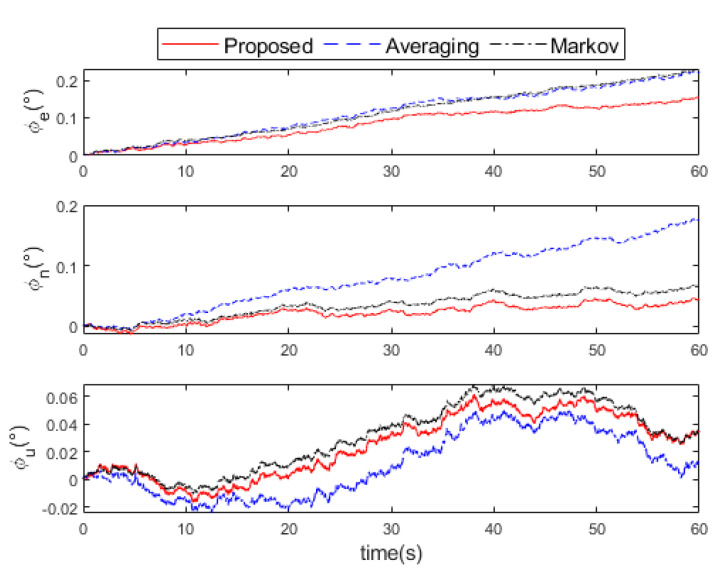
Attitude errors comparison of the proposed, averaging, and Markov methods in the experiment.

**Figure 9:**
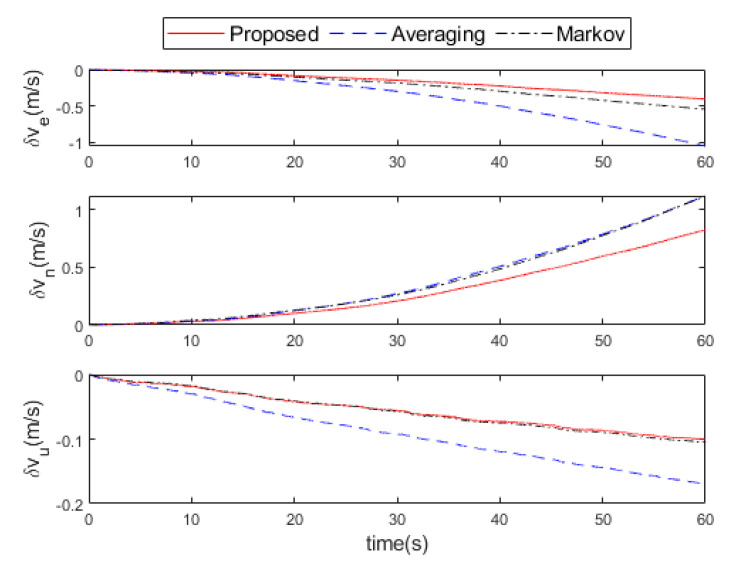
Velocity errors comparison of the proposed, averaging, and Markov methods in the experiment.

**Figure 10:**
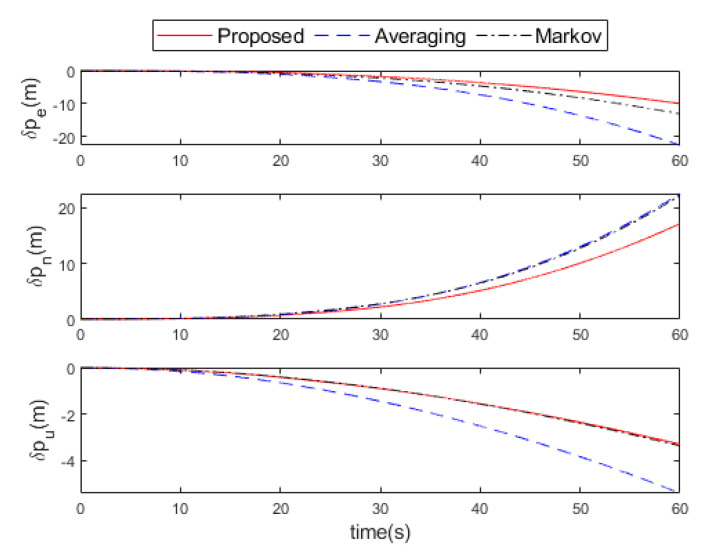
Position errors comparison of the proposed, averaging, and Markov methods in the experiment.

The attitude errors, velocity errors, and position errors after the fusion of the three methods are shown in Figure 8, Figure 9 and Figure 10, respectively. It can be seen that the proposed method is much smaller than the other two methods in the attitude errors of the east and the north direction. In the upward, the attitude error of the proposed method is similar to that of the Markov method, but both are slightly larger than the averaging method. However, in general, the attitude error of the proposed method is still minimal. After 60 s of navigation calculation, the velocity errors of the proposed method are (0.4,0.8,0.1) m/s in the east, north, and upward directions, which is better than (0.5,1.1,0.1) m/s by Markov method and (1.1,1.1,0.2) m/s by the averaging method. The position errors of the proposed method are (10,17,3) m in the east, north, and upward directions, which is better than (13,22,3) m by the Markov method and (23,22,5) m by the averaging method.

## 5. Conclusions

The error model of MEMS IMU has been established and the influence of random error on navigation has been analyzed in this study. To suppress random error and improve the navigation accuracy of low-cost MEMS IMU, the mechanism of random error suppression based on array technology has been studied. On this basis, a weighted averaging data fusion algorithm based on FOA is designed to meet the real-time requirements and further improve the accuracy of data fusion. The experiment shows that the proposed method can achieve higher navigation accuracy than the traditional methods, and then the effectiveness and superiority of the proposed fruit-fly-optimized weighted averaging algorithm are proved.

## Figures and Tables

**Table 1:** Stability of gyroscopes set in simulated MEMS IMU array.

Gyro	Gyro1	Gyro2	Gyro3	Gyro4	Gyro5
Stability (deg/h)	20	30	40	50	60

**Table 2:** Comparison of data fusion methods in simulation.

Method	Averaging	Markov	Proposed
Stability (deg/h)	19.02	14.35	14.35

**Table 3:** Comparison of data fusion methods in MEMS IMU array prototype.

Method	*x*-Axis (deg/h)	*y*-Axis (deg/h)	*z*-Axis (deg/h)
Averaging	7.65	8.88	6.22
Markov	6.78	5.55	5.10
Proposed	5.66	5.14	4.77

## Data Availability

The original contributions presented in this study are included in the article. Further inquiries can be directed to the corresponding author.
